# Diurnal Temperature Range is a Risk Factor for Coronary Heart Disease Death

**DOI:** 10.2188/jea.JE20080074

**Published:** 2009-11-05

**Authors:** Jingyan Cao, Yuexin Cheng, Ni Zhao, Weimin Song, Cheng Jiang, Renjie Chen, Haidong Kan

**Affiliations:** 1 Department of Cardiology, Fourth Affiliated Hospital of Nantong University, First Hospital of Yancheng, Yancheng, China; 2 Department of Hematology, Fourth Affiliated Hospital of Nantong University, First Hospital of Yancheng, Yancheng, China; 3 Department of Environmental Sciences and Engineering, School of Public Health, University of North Carolina at Chapel Hill, North Carolina, NC, USA; 4 School of Public Health, Fudan University, Shanghai, China; 5 Shanghai Municipal Center of Disease Control and Prevention, Shanghai, China

**Keywords:** diurnal temperature range, coronary heart disease, mortality, time-series, case-crossover

## Abstract

**Background:**

Although the relation between day-to-day temperature change and coronary heart disease (CHD) mortality is well established, it is unknown whether temperature variation within 1 day, ie, diurnal temperature range (DTR), is an independent risk factor for acute CHD death.

**Methods:**

We used time-series and case-crossover approaches to assess the relation between DTR and daily CHD mortality between 2001 and 2004 in Shanghai, China. Specifically, we used exposures averaged over periods varying from 1 to 5 days to assess the effects of DTR on CHD mortality. We estimated the percent increase in the number of daily deaths related to CHD that were associated with DTR, after adjustment for daily meteorologic conditions (temperature and relative humidity) and levels of outdoor air pollutants.

**Results:**

Both time-series and case-crossover analyses showed that DTR was significantly associated with the number of daily deaths related to CHD. A 1 °C increase in 2-day lagged DTR corresponded to a 2.46% (95% CI, 1.76% to 3.16%) increase in CHD mortality on time-series analysis, a 3.21% (95% CI, 2.23% to 4.19%) increase on unidirectional case-crossover analysis, and a 2.13% (95% CI, 1.04% to 3.22%) increase on bidirectional case-crossover analysis.

**Conclusions:**

Our findings suggest that DTR is an independent risk factor for acute CHD death.

## INTRODUCTION

Coronary heart disease (CHD) is a common cause of death and a leading cause of severe long-term disability in developed countries and some developing countries. Despite widespread concern and the great health burden imposed on middle-aged and elderly persons, associations between CHD and environmental risk factors have not been studied adequately.

Temperature affects human health. Global warming and other climate phenomena, such as El Nino, have sparked new interest in the weather–health relation.^1^^,^^2^ A relation between CHD mortality and temperature has been observed.^3^^–^^5^ A U-shaped relation between CHD mortality risk and temperature has been frequently reported, with a decrease in mortality from the lowest temperature up to an inflection point, after which the risk rises with increasing temperature. Of course, the relation varies with weather pattern, latitude, air pollution levels, and the prevalence of air-conditioning systems.^6^

Diurnal temperature range (DTR) is defined as the difference between the maximum and minimum temperatures in 1 day. Although a relation between temperature and CHD mortality has been observed,^3^^–^^5^ it is not known whether DTR is a risk factor for acute CHD death independent of the corresponding temperature. We hypothesized that a large DTR results in additional environmental stress to the human cardiovascular system, thereby increasing the risk for acute CHD death. We used time-series and case-crossover analyses of daily weather and mortality data from Shanghai, China to test our hypothesis.

## METHODS

### Data

Our study area included the 9 urban districts of Shanghai (total area, 289 km^2^): Huangpu, Jin’an, Luwan, Xuhui, Yangpu, Changnin, Yangpu, Putuo, and Zhabei. The target population was permanent residents living in the area: approximately 6.3 million people in 2000. Among the target population, the male/female ratio was 1.009, and 11.9% were older than 65 years.

The daily numbers of deaths related to CHD among residents living in the 9 urban districts of Shanghai from January 1, 2001 to December 31, 2004 were collected from the database of the Shanghai Municipal Center of Disease Control and Prevention (SMCDCP). In Shanghai, all deaths must be reported to the appropriate authorities before cremation. For the purpose of record-keeping, deaths among Shanghai residents occur in 2 locations: the hospital and the home. In both cases, the hospital or community doctors complete death certificates, after which the information on the certificates is sent to SMCDCP by means of an internal computer network. In the present study the causes of death were classified according to the International Classification of Disease (ICD), issued by the World Health Organization (WHO). CHD was defined as 390–398 and 410–429 in ICD9 in 2001, and as I00–I09 and I20–I52 in ICD10 in the period from 2002 through 2004.

Meteorologic data (daily minimum, maximum, and mean temperature; and relative humidity) were obtained from the Shanghai Meteorological Bureau. The weather data were measured at a fixed-site station located in the Xuhui District of Shanghai.

Air pollutants are potential confounders in a study of the association between DTR and CHD mortality.^7^^,^^8^ We therefore collected daily air pollution data from 2001 through 2004, including PM_10_, SO_2_, NO_2_, and O_3_ levels, from the Shanghai Environmental Monitoring Center.

### Statistical analysis

#### Time-series analysis

We used the generalized additive model (GAM) to analyze data on weather, CHD mortality, and air pollution. We first fitted nonparametric smoothing terms for trend on days, temperature, relative humidity (using a smoothing spline function), a dummy variable for days of the week (DOW), and a linear term for concentrations of air pollutants.^7^^,^^8^ Thereafter, DTR was introduced into the model. Because the assumption of linearity between the log of CHD mortality and DTR may not be justified, we used the smoothing function to graphically analyze their relation. We also considered the lag effects of weather conditions and pollutant concentrations in constructing the model. Residuals of each model were examined to determine whether there were discernible patterns or autocorrelations, by using residual plots and partial autocorrelation function plots.

It has been recently reported that the default convergence criteria in the widely used S-PLUS software *gam* function for implementation of the GAM do not assure convergence, and can provide biased estimates of both coefficients and their associated standard errors.^9^ Therefore, we used recently proposed, more stringent, convergence criteria.^9^ All analyses were performed using S-PLUS 7.0 (Seattle, WA) software. To avoid bias in the stand error of the coefficients, an extended GAM function was used to estimate the exact standard errors of the regression coefficients.^10^

#### Case-crossover analysis

Data analysis in case-crossover studies can be done by standard case-control methods. As originally formulated, an exposure that is temporally proximate to the event (case period) is compared with an exposure at a typical time when an event did not occur. In the present study, each CHD death was considered as a stratum, and the time at which a death occurred and another time were defined as the matched case and control periods, respectively. The association between CHD mortality risk and DTR was measured by odds ratios (ORs), using conditional logistic regression and the STATA statistical package (StataCorp, College Station, Texas). After controlling for mean temperature, relative humidity, and air pollutant concentrations, odds ratios were expressed for each 1 °C increment of DTR. Given that weather conditions may be nonlinearly related to CHD deaths, we used regression splines to control for temperature and humidity, each with 3 degrees of freedom.^11^

A number of control selection strategies have been proposed for the case-crossover design, and a detailed discussion of control sampling strategies has been published.^12^^,^^13^ For our study, we used both unidirectional retrospective control samplings and bidirectional control samplings. In the unidirectional retrospective control samplings, the same weekdays 1, 2, or 3 weeks before the death were selected as the control periods. For bidirectional control samplings the same weekdays 1, 2, or 3 weeks before and after the death were defined as the control periods. We chose the same weekdays as the control periods in order to avoid the day-of-the-week effect of mortality response. In addition, in constructing the models we also considered the lag effects of weather variables and pollutant concentrations on CHD mortality.

In both time-series and case-crossover analyses, we examined the effects of DTR for the warm season (April through September) and the cool season (October through March) separately.

## RESULTS

A total of 37 256 CHD deaths were reported to the SCDCP during our research period. This corresponded to an average of 25.5 deaths per day. The minimum, mean, and maximum DTR were 1.0 °C, 6.8 °C, and 16.6 °C during the same period (Table 1). Table 2 shows the correlations for daily values over the entire period for the weather and air pollutant variables. In general, DTR was significantly correlated with the concentrations of the 4 air pollutants we considered, and weakly correlated with mean temperature.

**Table 1.** Summary statistics for daily number of deaths related to coronary heart disease, meteorologic conditions, and concentrations of air pollutants in Shanghai, 2001–2004

	Mean	SD	Min	25th percentile	Median	75th percentaile	Max
CHD-related deaths	25.5	7.2	7.0	20.0	25.0	30.0	56.0
Meteorologic measures							
​ DTR (°C)	6.8	2.7	1.0	4.8	6.8	8.7	16.6
​ Mean Temperature (°C)	17.7	8.5	−2.4	10.3	18.3	24.7	34.0
​ Relative humility (%)	72.9	11.4	33.3	65.5	73.5	81.0	97.0
Concentrations of air pollutants							
​ PM_10_ (μg/m^3^)	101.9	64.4	14.0	56.0	84.0	128.0	567.0
​ SO_2_ (μg/m^3^)	44.7	24.2	8.0	27.0	40.0	56.0	183.0
​ NO_2_ (μg/m^3^)	66.6	24.8	14.0	50.0	62.0	79.0	254.0
​ O_3_ (μg/m^3^)	64.8	39.3	2.0	38.3	57.9	82.8	301.0

Abbreviations: CHD, coronary heart disease; DTR, diurnal temperature range; PM_10_, particulate matter <10 μm in diameter; SO_2_, sulfur dioxide; NO_2_, nitrogen dioxide; O_3_, ozone.

**Table 2.** Pearson correlation coefficients for meteorologic and air pollution variables in Shanghai, 2001–2004

	DTR	Mean temperature	Relative humidity	PM_10_	SO_2_	NO_2_	O_3_
DTR	1	0.13	−0.47	0.42	0.41	0.36	0.49
Mean temperature		1	0.21	−0.21	−0.20	−0.38	0.41
Relative humidity			1	−0.37	−0.52	−0.27	−0.36
PM_10_				1	0.63	0.70	0.19
SO_2_					1	0.73	0.17
NO_2_						1	0.06
O_3_							1

Abbreviations: DTR, diurnal temperature range; PM_10_, particulate matter <10 μm in diameter; SO_2_, sulfur dioxide; NO_2_, nitrogen dioxide; O_3_, ozone.

In both time-series and case-crossover analyses, we found a statistically significant association between DTR and the daily number of deaths related to CHD, after adjustment for long-term and seasonal trends in CHD mortality, DOW, temperature, relative humidity, and concentrations of air pollutants. Table 3 shows the adjusted percent increases in the daily numbers of deaths related to CHD, and 95% confidence intervals (CIs), associated with a 1 °C increase in DTR, using time-series (TS), unidirectional case-crossover (UCC), and bidirectional case-crossover (BCC) analyses. Models with 2-day lagged DTR produced the minimum AIC value in the time-series analysis: a 1 °C increase in DTR (lag = 2) corresponded to a 2.46% (95% CI, 1.76% to 3.16%) increase in CHD mortality on time-series analysis, a 3.21% (95% CI, 2.23% to 4.19%) increase on UCC analysis, and a 2.13% (95% CI, 1.04% to 3.22%) increase on BCC analysis. The estimated effects of DTR on CHD mortality were similar in the warm and cool seasons (Table 4).

**Table 3.** Estimated percent increase in the number of daily deaths related to coronary heart disease for each 1 °C increase in diurnal temperature range^a^

Lag	Method	Percent	95% CI
0	TS	1.12	0.68–1.56
	UCC	1.31	0.43–2.19
	BCC	0.70	0.18–1.22
1	TS	2.12	1.56–2.67
	UCC	2.67	1.41–3.93
	BCC	1.73	0.46–3.00
2	TS	2.46	1.76–3.16
	UCC	3.21	2.23–4.19
	BCC	2.13	1.04–3.22
3	TS	2.63	1.99–3.26
	UCC	3.62	2.09–5.15
	BCC	2.22	1.41–3.03
4	TS	2.60	1.95–3.26
	UCC	3.19	2.03–4.35
	BCC	2.37	1.12–3.62

Abbreviations: TS, time-series analysis; UCC, unidirectional case-crossover analysis; BCC, bidirectional case-crossover analysis.

^a^For UCC analysis, the same weekdays 1, 2, or 3 weeks before the death were selected as the control periods; for BCC analysis, the same weekdays 1, 2, or 3 weeks before, and after, the death were selected as the control periods.

**Table 4.** Percent increase in coronary heart disease mortality among Shanghai residents for each 1 °C increase in 2-day lagged diurnal temperature range, by season^a,b^

Method	Warm season	Cool season
TS	2.31 (1.74–2.88)	2.58 (1.96–3.20)
UCC	3.43 (2.01–4.85)	2.89 (1.66–4.12)
BCC	2.24 (1.61–2.87)	2.05 (1.07–3.03)

Abbreviations: TS, time-series analysis; UCC, unidirectional case-crossover analysis; BCC, bidirectional case-crossover analysis.

^a^For UCC analysis, the same weekdays 1, 2, or 3 weeks before the death were selected as the control periods; for BCC analysis, the same weekdays 1, 2, or 3 weeks before, and after, the death were selected as the control periods.

^b^Warm season, April through September; cool season, October through March.

**Figure 1.**
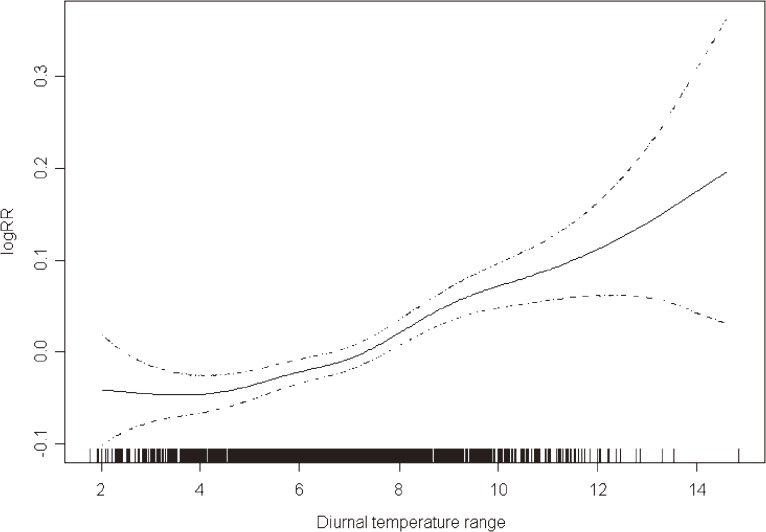
A smoothing plot demonstrating the association between DTR and CHD mortality (df = 5) on time-series analysis; the X-axis depicts DTR (°C). The estimated mean percent change in daily CHD mortality is shown by the solid line; the dotted lines represent pointwise twice-standard-error bands

Figure 1 shows the exposure–response relationships between DTR and CHD mortality at the best lagged-day (df = 5) in the time-series analysis. The associations were essentially linear for of the entire range of observed DTR values, which suggests that there is no threshold level for the association between DTR and CHD mortality.

In the case-crossover analysis, odds ratios varied in the different control schemes we used (Table 5). A model with 4 symmetric reference periods 7 and 14 days around the case period produced a similar but less statistically significant result. When only previous reference periods were used in the analysis, a larger effect was seen for DTR.

**Table 5.** Percent increase in number of daily deaths related to coronary heart disease for each 1 °C increase in 2-day lagged diurnal temperature range, by control scheme

Control scheme	Mean	95% CI
Before and after case		
​ 7 days	1.64	0.83–2.44
​ 7 and 14 days	2.10	1.37–2.83
​ 7, 14, and 21 days	2.13	1.04–3.22

Before case only		
​ 7 days	2.34	1.40–3.29
​ 7 and 14 days	2.44	1.63–3.25
​ 7, 14, and 21 days	3.21	2.23–4.19

## DISCUSSION

The acute effects of environmental factors such as ambient air pollutants (particulates, SO_2_, NO_2_, O_3_, and CO) and temperature (maximum, minimum, and mean) on CHD mortality have been well documented. Evidence from this study shows that temperature variation within 1 day—DTR—is also associated with the CHD mortality. Moreover, the association remained statistically significant after adjustment for temperature and other covariates, which suggests that DTR is an independent risk factor for acute CHD death. To our knowledge, this is the first study to report the acute effect of DTR on CHD mortality.

Although the underlying mechanism is unclear, studies have shown that sudden temperature changes might increase cardiovascular workload and induce the onset of a cardiovascular event.^14^^,^^15^ Regarding CHD deaths, we hypothesize that a large DTR results in additional environmental stress, and that stress on the cardiovascular systems increases during periods of considerable temperature change. In one human study, a sudden change in the temperature of inhaled air was associated with the release of inflammatory mediators associated with mast cells.^16^ In addition, most CHD deaths occur in elderly persons, among whom thermoregulatory capacity is often impaired^17^ and for whom sweating thresholds are generally higher than those of younger persons.^18^

Our study shows that the results of case-crossover studies greatly depend on the selection of control sampling strategies. Table 4 shows that the use of 4 and 6 controls before and after case periods provided results similar to those of the time-series study, and that the use of unidirectional retrospective control samplings resulted in a larger effect. As Navidi indicated, the results of unidirectional control sampling can be severely biased when time trends in exposure are strong, especially in the context of the relatively weak association between environmental exposure and health outcomes.^19^ To resolve such problems, he recommends the use of a bidirectional design in case-crossover analysis of exposure with time trends.^19^

There were some limitations in our analyses. The findings of studies on CHD incidence and CHD mortality differ somewhat. In our analysis, however, we were unable to differentiate CHD incidence from CHD mortality. Some studies have suggested that climate variables are associated with only some types of CHD. Due to limitations in the Death Registry System in Shanghai, we were unable to determine CHD subcategory. Like other similar studies, we used available environmental monitoring data to represent the population exposure to DTR and other covariates. In addition, it is uncertain whether air pollutants are confounders or effect modifiers (ie, whether a synergistic effect is present) of the association between DTR and CHD mortality. Future research might benefit from using an aggregate weather variable that incorporates temperature, temperature range (eg, DTR), relative humidity, dew point, wind speed, etc. Investigation of the effects of indoor air conditioning and heating systems on the association between weather and mortality should be also be conducted, given the wide use of such equipment in Shanghai.

In summary, although the association between climate change and DTR varies across the globe, and our findings regarding DTR and CHD mortality may therefore not apply to other areas, our data suggest that even a small increase in DTR is associated with a substantial increase in deaths due to CHD. By focusing on the risk factors for CHD mortality, eg, DTR, we might be able to significantly reduce CHD-related health problems. We believe that it is important to implement public health programs to prevent health problems due to temperature variation, even variation that occurs within one day. Of course, our findings require replication, especially in areas that differ in climate and air conditioning usage.
